# Caregiver Preferences regarding Technology's Role in Supporting Adolescent Weight Management

**DOI:** 10.1155/2015/153723

**Published:** 2015-11-26

**Authors:** Josette M. Bianchi-Hayes, Elinor R. Schoenfeld, Rosa Cataldo, Jiayu Huang, Susmita Pati

**Affiliations:** ^1^Department of Pediatrics, Stony Brook Children's Hospital and Stony Brook University, Stony Brook, NY 11794-8111, USA; ^2^Department of Preventive Medicine, Stony Brook University, Stony Brook, NY, USA

## Abstract

*Background*. Health technology provides a wealth of strategies to address chronic health issues, such as childhood obesity. Few studies have assessed parental preferences regarding use of health technology to support weight management for adolescents. *Objective*. This study determined caregiver beliefs, attitudes, and practices towards using traditional methods and technology-based health applications to address weight management among overweight adolescents. *Methods*. Self-administered surveys were distributed to caregivers of children ages 11–18 years in Stony Brook Children's Hospital outpatient offices with a BMI ≥ 85th percentile for age, gender. The data were entered into StudyTrax research platform and analyzed using SAS. *Results*.  *N* = 114. Mean BMI *z*-score = 1.95 ± 0.50. Two-thirds (65.8%) of caregivers preferred a weight management program that includes both traditional and technology components. Most parents rated involvement in program development (68.1%), access to content (72.4%) as very important. Those who believed their child's weight was a problem (*p* = 0.01) were more likely than other parents to prefer a program that combined both traditional and technology components. *Conclusions*. Parents' perceptions of their child's weight drove preferences about incorporating technology elements into a weight management program. Future weight management programs should incorporate parental content preferences and be tailored to different age groups.

## 1. Introduction

Childhood obesity has reached epidemic proportions within the United States with a prevalence of 16.9% in children aged 2–19 [1]. Capitalizing on previous literature, novel modalities for interventions have been employed, including family-based programs [2–4] and, more recently, interventions that incorporate technology [5–9]. Our study uses a caregiver/parent survey to better understand ways to combine the integral concept of family-centeredness with the ease, affordability, and novelty of technology in the preintervention stage of the development of a successful adolescent weight management intervention.

Several studies [2–4], as well as expert opinion [10], support the role of family in the process of weight management for children and adolescents. A 2012 American Heart Association position statement entitled “Evaluating Parents and Adult Caregivers as ‘Agents of Change' for Treating Obese Children,” found that parental/caregiver compliance with core behavioral strategies positively impacted their child's outcomes in studies related to weight management. However, the same paper highlighted research gaps in understanding the parental/caregiver impact on childhood obesity, including incorporation of new technologies and which parenting strategies are most effective [11]. Our study hopes to begin to bridge this gap through better understanding of ways to successfully incorporate technology into a family-centered intervention.

The emerging role of technology in both communication and self-monitoring provides a wealth of potential strategies to address complex and chronic health problems [12–16], like childhood obesity. Technology also can overcome some of the typical obstacles faced in implementing interventions such as cost, lack of adequate resources, and lack of time while addressing the importance of social support [17, 18]. Leveraging technology is particularly appropriate in the adolescent population, where 78% now have a cell phone with over half having a smartphone, and 23% have a tablet device [19].

Several studies have attempted to leverage technology, such as text messaging and smartphone applications, to address childhood and adolescent obesity [5–9]. Unfortunately, few mobile applications incorporate many of 2007 Expert Committee for Pediatric Obesity Prevention recommendations (e.g., reduce sugar-sweetened beverages) and few involved the families in the process [8].

By involving parents/caregivers from the preintervention foundational stages, we hope to develop an effective, family-oriented intervention that incorporates technology for adolescent weight management. This stage assessed the beliefs of the parents/caregivers, the practices families have already done to address weight loss, the beliefs and attitudes of parents toward technology use and supervision, and their beliefs and attitudes towards using technology as part of a weight management program. The caregiver perspective was the focus of this foundational stage of a multistage study which includes further data collection from both parents and adolescents in a variety of formats, including focus groups and a pilot intervention.

## 2. Materials and Methods

A self-administered survey for parents or caregivers of overweight and obese children 11–18 years of age was developed and prepiloted for content with parents and research staff. We obtained feedback from providers of pediatric weight management services, as well as experts in epidemiology, child health outcomes research, and behavior regarding the content and format of the questions in the survey. Paper based surveys were distributed at 5 Stony Brook Children's Hospital outpatient office sites (pediatric primary care, pediatric subspecialty, and pediatric weight management clinics) by trained research assistants during well child visits, acute visits, and weight management clinic visits. The data entry screen was developed with the assistance of the StudyTrax staff. The study was approved by the Stony Brook University Institutional Review Board prior to the start of study recruitment.

The survey was distributed to parents and legal caregivers of children ages 11–18 years who met weight requirements by gender and age. Adolescents were eligible to participate if they were overweight (BMI ≥85th percentile and <95th percentile for age and gender) or obese (BMI ≥95th percentile for age and gender) [20]. Additional eligibility criteria included the ability of the child to read and write in English, willingness of parent/caregiver to discuss issues related to the child's weight, the use of a technological element (smart phone, tablet device, or computer) by the child in the home, and the caregiver being the parent or legal guardian of the child. For the parent to participate, the child had to provide either written assent or consent (age-dependent).

114 surveys were completed by parents with associated adolescent anthropometric data gathered from the electronic medical record at the time of the physician visit. Nine surveys were started by a parent or caregiver but were deemed ineligible: 1 adolescent was too young, 3 adolescents had BMIs below eligibility criteria, 4 caregivers were not willing to discuss issues related to weight management, and 1 caregiver was not the legal guardian of the adolescent present for visit.

The survey administered to parents/caregivers included questions related to attitudes, beliefs, and practices with respect to healthy weight interventions, technology, and the combination of these elements for weight management (Appendix). Specific constructs included demographics, parental perception of weight (both as a categorical measurement and as a problem for their adolescent child), the practices their child has tried or is currently trying to lose weight, use of technology in the home (both device type (i.e., tablet device, smartphone) and supervision of use), and parental preferences for a technology-based weight management program for their adolescent child (including variables related to programming content, format, level of parental supervision, and involvement). Parents were also asked if they would be willing to have the child receive electronic messages (via text or email) from a weight management program and preferred times of the day to receive these messages. Lastly, parents were also asked whether they would be willing to have their child's weight issues discussed during hospitalization, to determine if this may be a feasible vehicle of recruitment for future weight management programs. Adolescent anthropometric data (height, weight, BMI, and BMI percentile) from the clinic visit was collected from the electronic medical record.

Data were entered into the StudyTrax research platform manually from paper copies of the survey by trained research assistants and manually cross-checked to ensure validity. The data were exported from StudyTrax into SAS for analysis. Frequency and cross tabulations were performed. Child's age, gender, and caregivers' perception of child's weight were primary predictors of interest. The covariants of child and caregiver race and ethnicity were not significant. Baseline category multinomial logistic regression models, as well as binary logistic regression models, were fitted to examine type of program caregiver desired (technology-based and/or traditional approaches). Since caregiver's ratings of intervention components were recorded as four-level Likert scale scores, cumulative logistic regression models for ordered outcomes were generated to assess what intervention elements parents most preferred. Both unadjusted and adjusted models were fitted.

## 3. Results

The demographics of study participants are presented in Table 1. A total of 114 caregivers/parents completed the survey. Approximately half of adolescents were female with a mean age of 13.6 ± 2.0. Age is presented as a categorical variable to highlight the important differences in the views and preferences of parents of younger (11–15 y/o) and older adolescents (16–18 y/o). The mean calculated BMI* z*-score of the adolescents was 1.95 ± 0.50. Most of the children in the study use computers (93.9%), the internet (96.5%), a smartphone (87.7%), and/or a tablet device (80%).

Parental perception of weight and actual weight is described in Table 2. Only 32.4% of parents correctly categorized their child as overweight, and 13.0% of parents correctly categorized their child as obese. Although 44.2% of all parents thought that their child's weight was a problem; only 13.5% of parents of overweight adolescents thought their child's weight was a problem (versus 59.2% for parents of obese adolescents).

The majority of parents (65.8%) envision a weight management program that includes both traditional and technology components. Among all parents surveyed, some parents (7.9%) would like to see only traditional components and a few parents (9.6%) would like to see only technological components in their child's weight management program. Furthermore, 16.7% of parents surveyed would like neither traditional nor technological components as parts of their child's weight management program. Parents most often rated healthy recipes (93.8%), exercise ideas (92.8%), and phrases of encouragement (87.8%) as important elements to include in a program. Those who believe their child's weight was a problem (OR = 4.41, 95% CI 1.34–14.56) were more likely than other parents to prefer both traditional and technology-based weight management programs to neither program (Table 3). Parents who believed their child's weight was a problem were significantly more likely than parents who did not think that their child's weight was a problem to want the following items in an intervention: electronic methods of tracking progress (OR = 3.91, 95% CI 1.68–9.07), communication with other program participants (OR = 2.64, 95% CI 1.29–5.40), a device to measure physical activity (e.g., Fitbit) (OR = 3.19, 95% CI 1.36–7.49), and exercise ideas (OR = 4.15, 95% CI 1.29–13.3) (Table 4(b)). Parents of older adolescents (16–18 years) were more likely to prefer a technology only-based program comparing all other options (traditional, traditional plus technology-based, or neither) (OR = 4.92, 95% CI 1.36–17.76) less likely to prefer a rewards-based program (i.e., electronic badges/stickers) (OR = 0.31, 95% CI 0.13–0.69) than parents of younger adolescents (11–15 years) (Tables 3 and 4(b)).

Parents in our study saw their role as important in both the development of the intervention and the supervision of the content. A majority (85.3%) of parents felt parental access to the technology used in a weight management program was either very or somewhat important. Additionally, 87.1% of parents felt their input into the development of the format/content of that program was very or somewhat important.

The survey assessed parental feelings about the weight management center contacting their adolescent via technology as part of an intervention; 89.4% of parents said that they felt very or somewhat comfortable having their children receive texts from a weight management program, and 89.4% responded similarly for email messages from the program. Lastly, 78.1% of parents responded that they would feel comfortable receiving messages after a discharge from the hospital regarding follow-up in the weight management center. We included this information as a way to assess potential, future recruiting mechanisms.

## 4. Discussion

To develop an effective family-oriented, technology-based intervention for adolescent weight management we surveyed parents/caregivers of overweight and obese adolescents to learn about their practices, perceptions, and preferences. This foundational step helped our research team better understand how an intervention can best incorporate the parent's views, as well as the parent themselves, into a successful intervention. We found that parents who believed their child's weight was a problem were significantly more likely than parents who did not believe this to (1) want electronic methods of tracking weight loss progress; (2) permit their child to communicate with other program participants; (3) incorporate a device to measure physical activity into the program; and (4) provide exercise ideas to their child. Those who believed their child's weight was a problem were more likely than other parents to prefer a program that incorporates both traditional and technology-based weight management techniques.

We also found that parents have preferences about the components of a weight management program for their adolescents. Parents expressed a desire to be involved in program development and have access to the program's content/devices used. Additionally, we found that the adolescent's age and the parental perceptions of their adolescent's weight were associated with some of their attitudes, beliefs, and preferences. Technology was more important for older and rewards were more important for younger adolescents.

The parent-child dynamic plays an important role in childhood obesity [21]. While previous literature shows that parental involvement in their child's weight management program plays a factor in their success, [11, 22–25] the current study is unique in that it provides empirical data that parents perceive their program involvement as important. These findings support the need for further discussions with parents/caregivers to more specifically define their optimal role in an adolescent weight management program. Including parents/caregivers in the program development process is supported by a prior study of obese preschool children that determined parental involvement in the design and implementation of a lifestyle intervention led to improvements in self-efficacy, rate of obesity, screen time, dietary intake, and light physical activity [26].

Despite the important role of the family for an adolescent to achieve weight loss success, only a small percentage (12.3%) of mobile applications that address pediatric obesity involve the whole family [8]. This example shows that there is a void in the market for family-based applications for children. Additionally, few studies have examined parental opinions of the role for technology in a weight management program for their children. Sharifi et al. explored parental perceptions of text messaging to support weight management. Through focus groups and follow-up interviews with 31 parents of 6–12-year-old children, they found that parents were open to their receiving text messages to support healthy behaviors for their children [27]. This study explored similar themes in parents of older children, within a larger sample size, and addressed a broader range of technologies.

Parents in our study reported that they envision a weight management program for their adolescent children to include both traditional and technology-based elements. Prior studies suggest that tailoring information to an individual provides for greater adherence rates, weight loss, and success for sustaining the weight loss [28, 29]. Incorporating technology to deliver tailored weight loss messages combined with a traditional weight loss program has the potential to reinforce the information delivered in a traditional program. Using technology as an outreach tool also has the potential to sustain and support the behavior change messages delivered during in-person provider visits.

We found that the majority of parents underreported their perceived adolescent's weight status, reporting their child as being either overweight or normal weight, regardless of calculated weight status. Previous studies, including a meta-analysis by Lundahl and an analysis of NHANES data by Chen [30, 31], support our findings of a disconnect between true anthropometrics and parental weight perception of their child. This inconsistency may serve as a barrier to seeking treatment for their child's weight issues as well as limiting parental involvement in a program. Future studies should address these potential barriers during both development and implementation of an adolescent weight intervention program.

Our study was limited to English-speaking parents/caregivers of children ages 11–18 years of age. We chose parents of this age range because we felt this was an appropriate age within which to explore the use of regular technology use. Parents of younger children may not be as comfortable with their child using technology regularly or communicating with others through technology. Additionally, our inclusion criteria limited our sample population to those who spoke English, eliminating some of our adolescent/caregiver dyads where one or both may have spoken another language, particularly Spanish. The questions were directed to the parent/caregiver in this stage of a more comprehensive study because we had planned to obtain adolescent perspectives through key informant interviews, focus groups, and a pilot intervention in later stages.

Another limitation of the study is the possible role of other socioeconomic factors as potential confounders. Our study did not explore family/household income or insurance status which may play an important role in caregiver perceptions regarding their adolescent's weight, access to technologies, and potential interventions. Since the study limited participations to families that already use some type of technology, results are not applicable to households with limited or no access to technology. Therefore, participants may be more likely to endorse use of each individual technology than other segments of the population.

While most of the patients in all of our clinical settings who are overweight or obese receive standard of care counseling on their weight, the large proportion of caregiver/adolescent dyads in the study recruited from the Pediatric Weight Management Center should be noted. These dyads may be different from those of overweight or obese status in the general pediatric or subspecialty clinics in terms of their motivation, level of interest, and perceptions.

## 5. Conclusion

The successful development of a family-oriented, technology-based weight management intervention for adolescents requires an understanding of parental opinions and preferences. This study further supports the concept of asking parents/caregivers to provide input and feedback for developing a technology-based adolescent obesity intervention. As a next step in development of an intervention, the study team will explore conducting a series of focus groups with parents/caregivers and adolescents to identify the key elements to include in the program while defining parental roles.

## Figures and Tables

**Table 1:** Demographics.

Characteristics	Total (*N* = 114)	
	Mean (SD)	Range
Child's weight		
Weight (kg)	81.8 (23.1)	[41.2, 188.5]
BMI	30.3 (6.4)	[21.7, 54.7]
BMI percentile	95.7 (4.1)	[85.2, 100.0]

	*N*	%

Child's weight category		
Overweight (≥85–95th percentile)	37	32.5
Obese (≥95th percentile)	77	67.5
Age category		
11–15 years	88	77.2
16–18 years	26	22.8
Gender		
Male	56	49.1
Female	58	50.9
Child's race		
White	80	69.0
African American	11	9.5
Other	24	20.7
Would rather not answer	1	0.9
Child's ethnicity		
Hispanic	29	25.0
Not Hispanic	82	70.7
Would rather not answer	5	4.3
Caregiver's race		
White	82	70.7
African American	10	8.6
Other	23	19.8
Would rather not answer	1	0.9
Caregiver ethnicity		
Hispanic	24	20.7
Not Hispanic	89	76.7
Would rather not answer	3	2.6

**Table 2:** Caregiver perception of child's weight versus child's actual weight.

			Child's actual weight status			
	Total		Overweight (≥85–95th percentile)		Obese (≥95th percentile)	
Total	114		37		77	

Caregiver believed child's weight was a problem	*N*	%	*N*	%	*N*	%
Yes	50	44.2	5	13.5	45	59.2
No	63	55.8	32	86.5	31	40.8
Missing	1	—	0	—	1	—
Caregiver thought child's weight was	*N*	%	*N*	%	*N*	%
Normal	38	33.3	25	67.6	13	16.9
Overweight	66	57.9	12	32.4	54	70.1
Markedly overweight	10	8.8	0	0.0	10	13.0

**Table 3:** Association between caregiver perception of child's weight, covariates, and type of program desired.

	Combined analysis of preference^a^			Prefer technology-based program *only* ^b^	Prefer traditional program *only* ^c^
	Technology, no traditional versus neither	Traditional, no technology versus neither	Both technology and traditional versus neither	Technology, no traditional versus others	Traditional, no technology versus others
Caregiver perception: believes child's weight is a problem					
No	1.0 (ref)	1.0 (ref)	1.0 (ref)	1.0 (ref)	1.0 (ref)
Yes	1.41 (0.25, 7.90)	1.88 (0.32, 11.02)	4.41 ****(1.34, ****14.56)^*∗*^	0.44 (0.11, 1.75)	0.61 (1.44, 2.56)
Child's age (years)					
11–15	1.0 (ref)	1.0 (ref)	1.0 (ref)	1.0 (ref)	1.0 (ref)
16–18	2.60 (0.56, 12.0)	1.73 (0.34, 8.87)	0.34 (0.11, 1.10)	4.92 ****(1.36, ****17.76)^*∗*^	2.98 (0.74, 12.05)
Gender					
Female	1.0 (ref)	1.0 (ref)	1.0 (ref)	1.0 (ref)	1.0 (ref)
Male	1.58 (0.34, 7.22)	0.72 (0.15, 3.54)	0.77 (0.28, 2.10)	1.97 (0.54, 7.14)	0.83 (0.21, 3.27)

^a^Baseline category multinomial logistic regression models were fitted. The reference category is “neither: caregiver wanted neither traditional nor technology components in a weight management program.”

^b^Binary logistic regression models were fitted. The reference category is “others: caregiver wanted ‘neither traditional nor technology components', or ‘both components', or ‘only traditional components' in a weight management program.”

^c^Binary logistic regression models were fitted. The reference category is “others: caregiver wanted ‘neither traditional nor technology components', or ‘both components', or ‘only technological components' in a weight management program.”

^*∗*^ *P* value < 0.05.

**(a):** 

	Healthy recipes	Exercise ideas	Encouragement phrases	Healthy images (photos of fruit, exercising)	Electronic methods to track progress	Rewards (i.e., electronic badges/ stickers)	Communication with others in the program	Device to measure physical activity
Caregiver perception: believes child's weight is a problem								
No	1.0 (ref)	1.0 (ref)	1.0 (ref)	1.0 (ref)	1.0 (ref)	1.0 (ref)	1.0 (ref)	1.0 (ref)
Yes	1.40 (0.61, 3.23)	**4.33 (1.35, 13.9)**	**3.06 (1.26, 7.46)**	**2.20 (1.08, 4.49)**	**4.06 (1.75, 9.37)**	1.92 (0.95, 3.89)	**2.78 (1.37, 5.70)**	**3.31 (1.42, 7.74)**
Child's age (years)								
11–15	1.0 (ref)	1.0 (ref)	1.0 (ref)	1.0 (ref)	1.0 (ref)	1.0 (ref)	1.0 (ref)	1.0 (ref)
16–18	0.65 (0.26, 1.65)	0.51 (0.18, 1.43)	0.61 (0.25, 1.51)	0.53 (0.24, 1.18)	0.66 (0.24, 1.31)	**0.29 (0.13, 0.66)**	0.47 (0.21, 1.06)	0.60 (0.25, 1.43)
Gender								
Female	1.0 (ref)	1.0 (ref)	1.0 (ref)	1.0 (ref)	1.0 (ref)	1.0 (ref)	1.0 (ref)	1.0 (ref)
Male	1.04 (0.46, 2.36)	1.15 (0.45, 2.95)	0.44 (0.19, 1.00)	0.84 (0.42, 1.67)	0.97 (0.46, 2.05)	0.89 (0.45, 1.76)	0.84 (0.42, 1.65)	(0.58, 2.74)

^a^All values are unadjusted odds ratios.

**(b):** 

	Healthy recipes	Exercise ideas	Encouragement phrases	Healthy images (photos of fruit, exercising)	Electronic methods to track progress	Rewards (i.e., electronic badges/ stickers)	Communication with others in the program	Device to measure physical activity
Caregiver perception: believes child's weight is a problem								
No	1.0 (ref)	1.0 (ref)	1.0 (ref)	1.0 (ref)	1.0 (ref)	1.0 (ref)	1.0 (ref)	1.0 (ref)
Yes	1.35 (0.58, 3.13)	**4.15** (1.29, ****13.3)^*∗*^	**2.97** (1.21, ****7.28)^*∗*^	**2.14** (1.04, ****4.38)^*∗*^	**3.91** (1.68, ****9.07)^*∗*^	1.79 (0.87, 3.65)	**2.64** (1.29, ****5.40)^*∗*^	**3.19** (1.36, ****7.49)^*∗*^
Child's age (years)								
11–15	1.0 (ref)	1.0 (ref)	1.0 (ref)	1.0 (ref)	1.0 (ref)	1.0 (ref)	1.0 (ref)	1.0 (ref)
16–18	0.68 (0.27, 1.72)	0.58 (0.20, 1.67)	0.68 (0.27, 1.71)	0.56 (0.25, 1.26)	0.64 (0.27, 1.55)	**0.31** (0.13, ****0.69)^t^	0.53 (0.23, 1.19)	0.68 (0.28, 1.67)

*P* value		0.02			0.002		0.008	0.008

^b^Caregiver's ratings of importance of intervention components were recorded in 4-level ordered Likert scale. Cumulative logistic regression models for ordered outcomes were fitted. Proportional odds assumption was checked for each model and none of them violated the assumption.

^*∗*^ *P* value = 0.01.

^t^ *P* value = 0.005.

## Appendix

(1) How old is your child?— —years (please list your child's age at their last birthday)

(2) Does your child read and write in English?

□ yes
□ no

(3) Are you willing to discuss your child's weight and topics related to your child's weight?

□ yes
□ no

(4) Do you or your child use a smart phone, tablet or computer?

□ yes
□ no

(5) Are you the parent or legal guardian of the child that you are here with today?

□ yes
□ no

If your child you are here with today is not between the ages of 11–18, or you answered no to any one of the questions above, we would like to thank you for your interest in this study but you do not meet the eligibility criteria at this time. Please give the electronic tablet and/or paper copies back to the research coordinator at this time. Thank you for your time.

(6) What is your* CHILD'S* month and year of birth? MONTH:— —YEAR:— — — —

(7) What is your child's gender?

□ male
□ female

(8) Are you Latino, Hispanic or of Spanish origin?

□ yes
□ no
□ would rather not answer

(9) Is your child Latino, Hispanic or of Spanish origin?

□ yes
□ no
□ would rather not answer

(10) What is your race?

□ White
□ Black, African American
□ Other
□ would rather not answer

(11) What is your child's race?

□ White
□ Black, African American
□ Other
□ would rather not answer

(12) Please tell us the reason for your child's visit today:

□ Physical exam, well visit
□ sick visit
□ Weight management clinic
□ Other

(13) How would you describe your child's current weight? (CHOOSE ONE)

□ markedly underweight
□ overweight
□ underweight
□ markedly overweight
□ normal

(14) Has any health care provider ever used any of the terms overweight or obese to describe your child?

□ yes
□ no

(15) Do you believe that your child's weight is a health problem?

□ yes
□ no

The next set of questions relate to your child's weight, nutrition and exercise patterns.

(16) What do you think your child's weight is?

□ — — —lbs.
□ do not know

(17) What do you think your child's height is?

□ —feet— —inches.
□ do not know

(18) Has your child* ever tried* any of the following programs or practices to address his/her weight? Check all that apply. If you do not know, please check “not sure.”

*Practice or Program*

Special diet

□ Ever tried
□ Never tried
□ Not sure

Formal exercise program

□ Ever tried
□ Never tried
□ Not sure

Keeping a food diary

□ Ever tried
□ Never tried
□ Not sure

Decreasing screen time (i.e. TV, computer)

□ Ever tried
□ Never tried
□ Not sure

Decreasing soda and juice

□ Ever tried
□ Never tried
□ Not sure

Smart phone or computer applications aimed at weight loss

□ Ever tried
□ Never tried
□ Not sure

Fitbit, pedometer or another personal electronic tracking device (a Fitbit is a wireless activity monitor that interfaces with a computer and/or Smartphone to track one's progress in fitness, nutrition, and sleep)

□ Ever Tried
□ Never tried
□ Not sure

(19) Please rate the following items on the scale below

1 = not a barrier to healthy living for your child
5 = a significant barrier to healthy living for your child

Available time to exercise

□ 1 (Not a barrier)
□ 2
□ 3
□ 4
□ 5 (A significant barrier)

Available safe place to exercise

□ 1 (Not a barrier)
□ 2
□ 3
□ 4
□ 5 (A significant barrier)

Cost of gym or sports participation

□ 1 (Not a barrier)
□ 2
□ 3
□ 4
□ 5 (A significant barrier)

Child's lack of interest in participation in exercise and/or physical activity

□ 1 (Not a barrier)
□ 2
□ 3
□ 4
□ 5 (A significant barrier)

Cost of healthy foods

□ 1 (Not a barrier)
□ 2
□ 3
□ 4
□ 5 (A significant barrier)

Personal knowledge of healthy recipes

□ 1 (Not a barrier)
□ 2
□ 3
□ 4
□ 5 (A significant barrier)

Lack of peer support for my child

□ 1 (Not a barrier)
□ 2
□ 3
□ 4
□ 5 (A significant barrier)

(20) How comfortable are you with your child using technology in the following ways (check one answer per statement)

*My Child's*

General use of internet websites

□ Very comfortable
□ Somewhat comfortable
□ Slightly uncomfortable
□ Very uncomfortable

General use of mobile text messaging

□ Very comfortable
□ Somewhat comfortable
□ Slightly uncomfortable
□ Very uncomfortable

Receiving messages geared toward health and wellness via text from a weight management program

□ Very comfortable
□ Somewhat comfortable
□ Slightly uncomfortable
□ Very uncomfortable

Receiving messages geared toward health and wellness via the internet (email or website message) from a weight management program

□ Very comfortable
□ Somewhat comfortable
□ Slightly uncomfortable
□ Very uncomfortable

Communicating with other children having similar issues with weight

□ Very comfortable
□ Somewhat comfortable
□ Slightly uncomfortable
□ Very uncomfortable

(21) Does your child use any of the following technology on at least an occasional basis? Is that technology supervised by a parent or caregiver? Check* ONE* for each technology.

Computer

□ Does not use at all
□ Uses ONLY with parent supervision
□ Uses ONLY without parent supervision
□ Uses with AND without parent supervision

Internet

□ Does not use at all
□ Uses ONLY with parent supervision
□ Uses ONLY without parent supervision
□ Uses with AND without parent supervision

Tablet (i.e. ipad)

□ Does not use at all
□ Uses ONLY with parent supervision
□ Uses ONLY without parent supervision
□ Uses with AND without parent supervision

TV

□ Does not use at all
□ Uses ONLY with parent supervision
□ Uses ONLY without parent supervision
□ Uses with AND without parent supervision

Smartphone (i.e. Droid or iphone)

□ Does not use at all
□ Uses ONLY with parent supervision
□ Uses ONLY without parent supervision
□ Uses with AND without parent supervision

(22) Which of the following would you like to see included in a weight management program for your child?

A traditional weight loss program (i.e. regular follow up with doctor and nutritionist)

□ yes
□ no

A program that incorporates modes of modern technology (i.e. in addition to doctor appointments, receive health-related text messages or participate in online peer support groups)

□ yes
□ no

(23) How important are each of the following areas to include in a technology-based weight management or wellness program for your child (i.e. website, text messaging program)?

Healthy recipes

□ Very important
□ Somewhat important
□ Slightly important
□ Not important

Exercise ideas

□ Very important
□ Somewhat important
□ Slightly important
□ Not important

Encouragement phrases

□ Very important
□ Somewhat important
□ Slightly important
□ Not important

Healthy images (i.e. photos of fruits or someone exercising)

□ Very important
□ Somewhat important
□ Slightly important
□ Not important

Electronic methods to track progress

□ Very important
□ Somewhat important
□ Slightly important
□ Not important

Reward concepts (i.e. electronic badges/stickers)

□ Very important
□ Somewhat important
□ Slightly important
□ Not important

Communication with others in the program

□ Very important
□ Somewhat important
□ Slightly important
□ Not important

Device to measure physical activity (i.e. Fit Bit)

□ Very important
□ Somewhat important
□ Slightly important
□ Not important

(24) What forms of supervision do you feel are important to incorporate into a technology-based weight management and wellness program for your child (i.e., website, text messaging)?

Parental access to devices or websites used in program

□ Very important
□ Somewhat important
□ Slightly important
□ Not important

Discussion with your child about their progress and their experience

□ Very important
□ Somewhat important
□ Slightly important
□ Not important

Parental input involved in developing the format and content of program

□ Very important
□ Somewhat important
□ Slightly important
□ Not important

(25) Please list other forms of supervision that you feel are important to incorporate into a technology-based weight management and wellness program for your child?

□ none

(26) The best time of day for my child to receive messages and/or communication through technology (i.e., text messages, internet messages) is (CHECK ONE):

□ Early am (6:00 am–8:00 am)
□ Morning (8:01 am–12:00 pm)
□ Early afternoon (12:00 pm–2:00 pm)
□ Late afternoon (2:01 pm–5:00 pm)
□ Evening (5:01 pm–7:01 pm)

(27) If your child was ever admitted to the hospital, when would be the best time to discuss issues related to weight with your child? (CHECK ONE):

□ During hospitalization
□ After discharge
□ I would not like to discuss weight issues

(28) If your child was ever admitted to the hospital, would you be comfortable receiving messages after discharge from the hospital regarding follow-up with a Pediatric weight management center?

□ yes
□ no

*Research Assistant Enters from EMR OR Chart*

Weight: — — —.— —kg

Height: — — —.—cm

BMI: — —.— —

BMI percentile: — — —.— —%

## Abbreviations

BMI: Body mass index
CDC: Centers for Disease Control and Prevention
SAS: Statistical Analysis System
CI: Confidence interval.
